# Gene Therapy for Human Sensorineural Hearing Loss

**DOI:** 10.3389/fncel.2019.00323

**Published:** 2019-07-16

**Authors:** Yin Ren, Lukas D. Landegger, Konstantina M. Stankovic

**Affiliations:** ^1^Department of Otolaryngology, Harvard Medical School, Boston, MA, United States; ^2^Eaton Peabody Laboratories, Department of Otolaryngology, Massachusetts Eye and Ear, Boston, MA, United States; ^3^Department of Otolaryngology, Vienna General Hospital, Medical University of Vienna, Vienna, Austria; ^4^Program in Speech and Hearing Bioscience and Technology, Harvard Medical School, Boston, MA, United States; ^5^Harvard Program in Therapeutic Science, Harvard University, Boston, MA, United States

**Keywords:** gene therapy, adeno-associated virus (AAV), nanoparticles, blood labyrinth barrier, Anc80L65, tumor penetrating peptide, round window niche

## Abstract

Hearing loss is the most common sensory impairment in humans and currently disables 466 million people across the world. Congenital deafness affects at least 1 in 500 newborns, and over 50% are hereditary in nature. To date, existing pharmacologic therapies for genetic and acquired etiologies of deafness are severely limited. With the advent of modern sequencing technologies, there is a vast compendium of growing genetic alterations that underlie human hearing loss, which can be targeted by therapeutics such as gene therapy. Recently, there has been tremendous progress in the development of gene therapy vectors to treat sensorineural hearing loss (SNHL) in animal models *in vivo*. Nevertheless, significant hurdles remain before such technologies can be translated toward clinical use. These include addressing the blood-labyrinth barrier, engineering more specific and effective delivery vehicles, improving surgical access, and validating novel targets. In this review, we both highlight recent progress and outline challenges associated with *in vivo* gene therapy for human SNHL.

## Introduction

Hearing loss is the most common sensory impairment in humans and currently disables 466 million people across the world; this number is expected to rise to nearly 1 billion by 2050 (WHO Deafness and Hearing Loss, 2018). It is especially prevalent in the aging population as nearly two-thirds of the U.S. population over the age of 70 years are affected by disabling hearing loss (Lin et al., 2011). Furthermore, congenital deafness affects at least 1 in 500 newborns, with over 50% of these being hereditary in nature. Most of this burden is due to sensorineural hearing loss (SNHL) which originates from defects in the cochlea, the spiraling organ of the inner ear.

The human inner ear is a small, three-dimensionally complex, fluid-filled structure encased in the densest bone in the body and located deep in the base of skull. Acoustic energy from sound is transmitted to the fluids of the cochlea via vibrations of the tympanic membrane and ossicular chain in the middle ear, producing a traveling wave along the basilar membrane. The length of the cochlea and stiffness of the basilar membrane enables the differentiation of sound frequencies (Manoussaki et al., 2006). This in turn leads to activation of mechanotransduction by hair cells, specialized sensory cells located in the organ of Corti, which turn mechanical stimulation into electrical depolarization. The electrical signal initiated by the inner hair cells (IHCs) is then processed by spiral ganglion neurons (SGNs) that make up the auditory nerve and ultimately decoded in the auditory cortex of the temporal lobe (Rubel and Fritzsch, 2002).

The genetic basis for human hearing loss has been under intensive investigation for the past two decades. Initially noted by Gorlin et al. (1995) that a significant portion of hearing loss has an underlying genetic etiology, the number of distinct genes associated with inherited hearing loss has since rapidly expanded with the advent of advanced sequencing technologies. A list of human loci linked with hearing loss has been compiled and regularly updated^1^. Broadly speaking, genetic hearing loss is subcategorized into Mendelian inheritance including both syndromic and non-syndromic cases, or complex inheritance which includes both genetic and environmental factors. Today, there are 115 genes responsible for non-syndromic hearing loss, with 45 autosomal dominant genes, 73 autosomal recessive genes, 5 X-linked genes, and additional loci for modifiers, Y-linked, and auditory neuropathy, respectively. On a global scale, the prevalence of hearing loss is highest in Eastern Europe, Central and South Asia, and Asia Pacific approaching 10%. Regions with lower income and literacy levels also tend to have higher rates of hearing loss (Sheffield and Smith, 2018). Further characterizations of the molecular pathways defined by these genes and loci have been reviewed elsewhere (Dror and Avraham, 2010; Stamatiou and Stankovic, 2013).

Despite our growing knowledge of the molecular underpinnings of auditory development, as well as an expanding armamentarium of deafness genes identified to date, there are no pharmacotherapies clinically approved for SNHL. Current treatments focus on amplification of sound through hearing aids, or via electrical stimulation of auditory neurons through cochlear implantation (CI) for severe to profound deafness. Nevertheless, neither approach restores the native inner ear sensory epithelium. Given the genetic basis underlying many forms of hearing loss and progress in our understanding of the mechanisms of hair cell regeneration and neuronal synapse repair, targeted modulation of affected genes in specific cell types of the inner ear could be a powerful therapeutic strategy. There have been numerous recent reports highlighting the complexity of genetic hearing loss and both non-viral and viral delivery approaches for therapeutic delivery (Chang, 2015; Ahmed et al., 2017; Devare et al., 2018; Lustig and Akil, 2018). In this review, we focus on (1) transport barriers to inner ear drug delivery, (2) viral and nanotechnology carriers that target the inner ear with high precision and efficiency, (3) adult animal models for clinically translatable hearing restoration, and (4) practical surgical challenges in navigating the middle and inner ear.

## Transport Barriers to the Inner Ear

**Figure 1:**
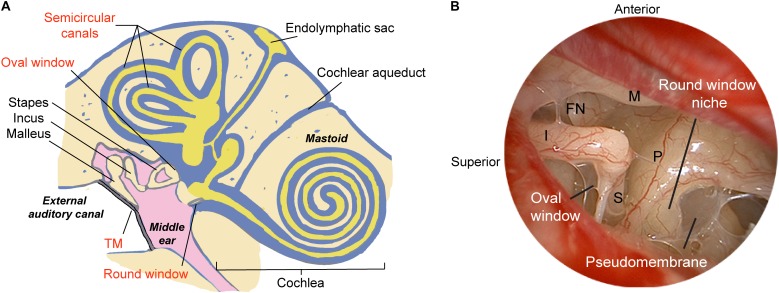
Schematic illustrating methods of delivering therapeutics to the human ear. **(A)** The relevant structures through which drugs such as gene therapy agents, nanoparticles, or biologics are introduced into the inner ear are labeled in red, and include both indirect and direct approaches. The indirect approach is through the tympanic membrane (transtympanic or intratympanic) to deposit the therapeutic in the middle ear and allow it to diffuse into the inner ear via the oval and round windows. Direct approaches include delivery into the cochlea through application over or through the round window membrane, through a surgically drilled cochleostomy adjacent to the round window, a fenestra in the bony oval window, or a semicircular canal. Approaches for drug/gene delivery also include combining existing technologies such as cochlear implant electrodes or stapes prosthesis during stapedotomy. **(B)** Endoscopic view of the anatomy of the human middle ear. The round window niche consists of bony overhang, and the round window is often obscured by a pseudomembrane. M, malleus manubrium; I, incus; P, cochlear promontory; S, stapes; FN, facial nerve; TM, tympanic membrane. Image reproduced with permission from Medscape Drugs & Diseases (https://emedicine.medscape.com/), Surgical Treatment of Meniere Disease, 2018, available at: https://emedicine.medscape.com/article/856658-overview.

The translation of modern molecular therapy into clinical use is hindered by the so-called “delivery” challenge. To engineer a precise and effective inner ear delivery system, one must first develop a comprehensive understanding of the anatomic and physiologic barriers that isolate labyrinthine organs from the middle ear space and the brain. The perilymphatic space communicates via the round and oval windows to the middle ear, and via the cochlear aqueduct and cochlear modiolus to the cerebral spinal fluid (CSF) space (Figure 1). The arterioles and venules are located inside bony channels within the scala vestibuli and scala tympani, respectively, with the major capillary beds located in the stria vascularis, spiral ligament, and spiral ganglion (Axelsson, 1988). A lymphatic system is also thought to provide clearance and drainage of the middle and inner ear (Lim and Hussl, 1975). On a microscopic scale, specialized cell layers consisting of tight junctions and endothelial cells lining cochlear blood vessels form the blood-labyrinth barrier (BLB), an intricate network that tightly regulates the transport of macromolecules and ions between the vascular compartment and the inner ear.

The BLB has been compared to the blood–brain barrier (BBB), a complex system of endothelial cells, basement membrane, pericytes, and astrocytes that isolates and protects the central nervous system (CNS). While the complexity of the BBB renders delivery of macromolecule therapeutics challenging, it also presents unique opportunities for drug delivery across the barrier (Pardridge, 2016). Novel ways to improve therapeutic delivery include modifications of the drug to improve pharmacokinetics and lipophilicity, development of “Trojan horse” carriers or analogs for endogenous ligands, inhibition of drug efflux, and modulation of BBB permeability (Banks, 2016). Recent promising work in nanotechnology has overcome this bottleneck and bypass the BBB in pathologic conditions such as traumatic brain injury (Smith et al., 2016). These promising strategies should be emulated and adapted to enhance drug delivery across the BLB into the inner ear.

Under physiologic conditions, the intact BLB is thought to maintain ionic homeostasis, isolate from pathogens in the blood, and thereby contribute to the semi immune-privileged nature of the inner ear (Salt and Hirose, 2018). By contrast, this barrier can become “leaky” upon exposure to toxic levels of noise or ototoxic medications through activated macrophages (Kopke et al., 1999; Suzuki et al., 2002). Interestingly, evidence suggests that a compromised BLB through systemic exposure to lipopolysaccharide does not correlate with a loss of hearing (Hirose et al., 2014; Hirose and Li, 2019). Therefore, the dynamic nature of the BLB’s permeability could be harnessed as a window for intracochlear therapeutic delivery. Indeed, strategies to temporarily enhance the permeability of tissue barriers have seen success in delivering macromolecule drugs to tumors or the brain that would be otherwise difficult to traverse (Ren et al., 2012; Kwon et al., 2016). Future experimental animal models will need to specifically and precisely alter the permeability of the BLB and identify new epitopes that are unveiled in the cochlear sensory epithelium or neurons. Strategies to reduce lymphatic or macrophage-mediated clearance of delivered drugs could effectively improve the biodistribution of the drug within the desired tissue. Future interventions that transiently enhance macromolecule transport across the BLB without causing significant cellular damage may serve as useful adjuncts to improve drug penetration.

## Model Systems for Gene Therapy

While optimization and characterization of gene therapy vectors could be done in cell cultures and cochlear explants *in vitro*, animal studies are required to understand the full panoply of the gene’s effects in the inner ear microenvironment. Furthermore, the long-term therapeutic effects, interactions with other organ systems and organ toxicities can only be characterized in a living host. Historically, *in vivo* gene delivery experiments have been carried out in zebrafish, birds, and rodents. Both zebrafish and birds have well-characterized genomes and robust regenerative capacity in the inner ear sensory epithelium after injury (Daudet et al., 2009; Cotanche and Kaiser, 2010); nevertheless, these non-mammalian model systems are rarely utilized for gene transfer research due to dissimilarities from mammals and humans.

Rodents are the preferred animal models due to their well-characterized genomes, robustness to manipulations, cost-effectiveness, and close resemblance to the human inner ear and potential for pre-clinical testing. While the human inner ear is larger than that of rodents, the cochlea of rodents such as guinea pigs can be more accessible due to their unique anatomy where the otic capsule protrudes into the middle ear space and contains thin bony walls (Mikulec et al., 2009; Jawadi et al., 2016). Today, transgenic mouse models capturing various genetic alterations that mimic human disease are well characterized, and mutant mice carrying simultaneous mutations in multiple genes crucial for normal hearing function have been established using CRISPR/Cas-9 technology (Zhang et al., 2018). However, experiments involving delivery of gene therapy using novel AAV systems to the cochlea have been performed largely in neonatal mice (Landegger et al., 2017; Pan et al., 2017). In a mouse model of hereditary deafness through a null mutation in the vesicular glutamate transporter-3 (VGLUT3) responsible for IHC-afferent nerve synaptic transmission, early intervention using AAV-mediated gene delivery at postnatal days 1–3 led to more efficient and longer duration of hearing recovery than intervention at postnatal day 10 (Akil et al., 2012). While these results are exciting, they may have limited potential for direct clinical correlation and translation to treatments of newborns and adult patients with hearing loss.

Unlike humans, mice are born deaf and begin to hear at approximately 2 weeks of age. Therefore, delivery of therapeutics to neonates would be equivalent to *in utero* therapy in humans, and thereby pose additional significant technical challenges. In mouse models of progressive hearing loss, gene therapy is typically administered prior to organ of Corti beginning to degenerate in the early postnatal period. By contrast, in many forms of hereditary deafness, there is likely already degeneration of the sensory epithelium and neurons which occurred early *in utero*, thereby rendering postnatal therapy targeting either hair cells or SGNs futile.

To address these concerns, recent reports utilizing common AAVs, Anc80L65, and 7m8 vectors showed efficient transduction of vestibular hair cells in adult mice ranging from P15 to P60, without jeopardizing their cochlear function (Suzuki et al., 2017; Yoshimura et al., 2018; Isgrig et al., 2019). Therefore, future studies should continue to focus on expanding the therapeutic window and developing cochlear gene therapies for adult-stage hearing loss, which would be equivalent to the clinical scenario of treating patients with hearing loss months to even years after the initial insult.

Prior to conducting human clinical trials, it is also critically important to test the efficacy and safety of hearing-restoration therapeutics in non-human primates. Inner ear volumes (including soft tissue and fluid) correlate with body mass across species, with an estimated volume of 2.3–2.5, 59.4–63, and 191.1–237 μL for mouse, rhesus monkey, and human, respectively (Ekdale, 2013; Dai et al., 2017). While it is tempting to apply results from mouse studies to human clinical trials, the large discrepancies in inner ear volumes may make such direct extrapolations challenging. Since the human cochlea and labyrinth measures approximately two orders of magnitude larger than that in rodents and only three- to fourfold larger than monkeys, it may be easier to extrapolate successful technical implementation of gene delivery in rhesus monkeys to humans. To achieve this goal, Dai et al. (2017) assessed rhesus audiovestibular functions after mock saline injections via either the oval or round windows. Encouragingly, nearly all animals tolerated the injections with no evidence of toxicity in histological, audiometric, and behavioral analyses (Dai et al., 2017). Based on these results, György et al. (2019) performed the first transmastoid injection of an AAV9 variant via the round window membrane (RWM) in a cynomolgus monkey, which appeared to mediate efficient transduction of both inner and outer hair cells (György et al., 2019). Nevertheless, results were inconsistent as a second monkey showed significantly limited transduction at a lower dose. Future studies utilizing a larger number of animals are required to understand variables such as pre-existing immunity to the AAV vector (Louis Jeune et al., 2013), the dose-dependency of transduction efficiency, and the technical reproducibility of RWM injections in larger mammals (György et al., 2019).

## Vehicles and Targets

Nearly half of all congenital hearing loss arises from genetic factors, and approximately two-thirds of these are inherited. Most non-syndromic deafness is autosomal recessive (75–90%) with mutations in *GJB2* and *SLC26A4* responsible for the majority of cases; most of the remaining cases are inherited in an autosomal dominant pattern, and a small fraction of up to 1.5% are either X-linked or mitochondrial (Dror and Avraham, 2010; Stamatiou and Stankovic, 2013; Chang, 2015). Compared to gene therapy for acquired hearing loss after mechanical or pharmacological insults, genetic hearing loss poses greater challenges in that successfully regenerated hair cells or neurons would still harbor the causative genetic defect. Therefore, targeted correction of the underlying genetic mutation in all affected cell populations in the cochlea is paramount. Fortunately, with advent in modern sequencing technologies and prenatal testing, it is now becoming possible to detect genetic defects and intervene on them at an earlier stage.

The choice of delivery vector for *in vivo* gene therapy depends on many factors including the cell type being targeted, route of administration, and therapeutic potency. Delivery platforms can be broadly classified into viral vectors including adenoviruses (AdVs), adeno-associated viruses (AAVs), and retroviruses including lentiviruses (Kiernan and Fekete, 1997; Pietola et al., 2008); versus non-viral vectors such as nanoparticles and exosomes. In theory, viral vectors generally enable more stable and durable expression of the transgene. However, the complex immune response to viruses and the safety of long-term transgene expression are unknown and can be challenging to assess. By contrast, non-viral delivery vehicles are less immunogenic and can be engineered to satisfy the exact therapeutic need. A summary of the viral and non-viral vectors reported in the literature over the last 10 years for *in vivo* delivery in mature animals is shown in Table 1.

**Table 1:** A summary of the viral vectors recently reported in the literature in the last 10 years for *in vivo* gene delivery in adult animals.

Model (References)	Age	Follow-up	Sex (*n*)	Vector(s)	Route	Outcome
Guinea pig (pigmented) (Shibata et al., 2009)	N/A	2 weeks	*M* (*n* = 20)	BAAV-CMV–β-actin–GFP	Cochleostomy	Cochlea: Transduced the supporting cells of both normal and deafened animals
Mouse (CBA/CaJ) (Kilpatrick et al., 2011)	2–12 months	5 months	M&F (*n* = 120)	AAV2/1-CMV-EGFP (also AAV2/2, 5, 6, 8)	Cochleostomy	Cochlea: Efficient AAV inoculation (via the scala media) can be performed in adult mouse ears, with hearing preservation
Guinea pig (pigmented) (Budenz et al., 2015)	1–2 months	3 months	M&F (*n* = 46)	AAV2/2-CBA-EGFP, AAV2/2-CBA-NTF3, AAV2/2-CBA-BDNF	Cochleostomy	Cochlea: Transient elevation in neurotrophin levels can sustain the cochlear neural substrate in the long term
Mouse (CBA/J) (Chien et al., 2015)	1–2 months	4 weeks	M&F (*n* = 66)	AAV2/8-CMV-GFP	Cochleostomy and RWM	Cochlea: Cochleostomy and RWM approach can both be used. The RWM approach results in less hearing loss vs. cochleostomy
Mouse (CBA/CaJ) (Shu et al., 2016)	6 weeks	3 months	M (*n* = 4)	AAV2/1-CAG-EGFP (also AAV2/2, 5, 6, 6.2, 7, 8, 9, rh.8, rh.10, rh.39, rh.43)	Cochleostomy	Cochlea: AAV1, 2, 6.2, 7, 8, 9, rh.39, rh.43 transduced IHCs, but no OHCs – even partial OHC loss.
Guinea pig (pigmented) (Lee et al., 2016)	1–2 months	3 weeks	M&F (*n* = 26)	Ad5-Empty, Ad5-NTF3, AAV2/2-CBA-NTF3	Cochleostomy	Cochlea: Hearing threshold shifts, disorganization of peripheral nerve endings, and synaptic disruption with both vectors. Elevation of NT3 levels in cochlear fluids can disrupt innervation and degrade hearing.
Mouse (CBA/CaJ) (Suzuki et al., 2017)	7 weeks	2 weeks	M (*n* = 13)	AAV2/Anc80L65-CASI-EGFP-RBG	PSCC	Cochlea: Successful transduction of all IHCs, majority of OHCs especially at apex, and 10% of SGNs. Vestibular: Maculae and cristae transduced. Transduction of many hair cells, all supporting cells.
Mouse (C57BL/6) (Gao et al., 2018)	6 weeks	2 weeks	M&F (*n* = 3)	Cas9:GFP sgRNA:lipid complex	PSCC	Cochlea: Target gene disruption at 25 ± 2.1% efficiency, i.e., probably applicable to dominant genetic deafness manifested with late-onset hearing loss
Mouse (C57BL/6J and CD1) (Tao et al., 2018)	8–10 weeks	7 weeks	M (*n* = 29)	AAV2/1-CAG-EGFP (also AAV2/2, 6.2, 8, 9, rh.39, rh.43), AAV2/Anc80L65-CMV-EGFP-WPRE, Ad5-CMV-EGFP	PSCC	Cochlea: Most AAVs transduce IHCs efficiently, but are less efficient at transducing OHCs. Subset of AAVs transduces other cell types. Canalostomy can be a viable delivery route.
Mouse (FVB/N) (Guo et al., 2018)	5–6 weeks	1 week	F (*n* = 1)	AAV2/8-GFP	PSCC	Canalostomy is an effective and safe approach to drug delivery into the inner ears of adult mice.
Mouse (C57BL/6) (György et al., 2019)	4 weeks	2 weeks	M&F (*n* = 2)	AAV2/9-PHP.B-CBA-GFP	PSCC	Cochlea: Almost all IHCs from apex to base transduced, no OHC transduction. Vestibular: Robust transduction.
Mouse (C57BL/6J) (Nist-Lund et al., 2019)	4 weeks	7 weeks	M&F (*n* = 12)	AAV2/Anc80L65-CMV-TMC1-WPRE, AAV2/Anc80L65-CMV-TMC2-WPRE, AAV2/Anc80L65-CMV-TMC1EX1-WPRE, AAV2/Anc80L65-CMV-EGFP-WPRE	RWM	Cochlea: Gene therapy rescue of sensory function in mature hair cells. Vestibular: Gene therapy recovery of balance even possible at mature stages.
Mouse (CBA/J) (Isgrig et al., 2019)	1–6 months	4 weeks	M&F (*n* = 6)	AAV2/7m8-CAG-EGFP	PSCC	Cochlea: Successful transduction of IHCs (84.5%) and OHCs (74.9%). Vestibular: Only data for neonatal animals – less efficient in vestibular organs than cochlea.

*In case of neonatal and adult injections in the same study, only the adult ages are mentioned in the table. If the n for a specific experiment wasn’t explicitly stated, figures were analyzed to infer the minimal n (listed). When no sex was mentioned, it was assumed that male and female animals were used. When detailed vector information was not available, the listed vector in the table was assumed based on other information in a given paper or information provided by a study author who was individually contacted*.

### Viral Delivery Agents

Much of the pre-clinical success in gene therapy for hearing loss is owing to the use of viral vectors to carry payloads into the inner ear. Popular viral vectors include variants of AdV and AAV, both of which have excellent tropism toward a number of cell types in the cochlea and relatively high transduction efficiency in animal models (Akil et al., 2012; Askew et al., 2015). AdV-mediated delivery results in transient expression of the transgene, whereas AAV-mediated delivery could lead to long-lasting gene expression. A summary of the advantages and disadvantages of both AdV and AAV vectors for cochlear gene therapy have been the topics of many recent reviews (Husseman and Raphael, 2009; Ahmed et al., 2017; Lustig and Akil, 2018).

Of note, a particular synthetic “ancestral” AAV subtype, Anc80L65, was able to efficiently transduce over 90% of both inner and outer hair cells at a dose that is two to three times lower than conventional AAV counterparts (Landegger et al., 2017). In a mouse model of Type I Usher syndrome due to mutations in *Ush1c* encoding the protein harmonin, wild-type harmonin was successfully delivered into the inner ear after RW membrane injection using the Anc80 vector. In neonatal P0-1 mice, the delivered gene product was found to be localized to the stereocilia near tip-link insertions on hair cells. Ultrastructural studies using scanning electron microscopy showed normal hair cell morphology and decreased hair cell loss. The treatment not only restored mechanotransduction, but also led to dramatic improvements in complex audiovestibular functions to near wild-type levels for at least 6 months (Pan et al., 2017). These results suggest that virally mediated expression of the transgene can rescue hair cell function if they are present before hair cells degenerate as the animal matures, and such effects could be long-lasting for the duration of development.

Another unresolved question in viral-mediated cochlear gene therapy is whether stable levels of transgene expression can be sustained, and if so, for how long. Since many genes implicated in human deafness are only transiently expressed during normal development, how severe are the negative effects from over-expression? As cells in the sensory epithelium do not typically divide, expression of exogenously transduced genes is not diluted from cell division and could theoretically remain stable over time (Bainbridge et al., 2015). Previous studies in mice have shown stable treatment efficacy with follow-ups of 3–6 months (Akil et al., 2012; Chang et al., 2015; Isgrig et al., 2017; Pan et al., 2017), but few studies have examined the phenotype after this time point.

A related field where rapid progress in gene therapy has occurred in the last decade is the treatment of inherited retinal dystrophies. Importantly, the first successful clinical application of gene therapy involved treatment of inherited blindness (Dalkara et al., 2016). AAV-mediated delivery of *RPE65* gene for treatment of Leber’s congenital amaurosis received FDA approval in January 2018. This represented the first directly administered clinical gene therapy in the United States that targets a disease caused by mutations in a single gene. Interestingly, long-term studies in patients who underwent gene therapy showed a decline in vision improvement at the 3-year time point, possibly due to the decline in RPE65 expression below a certain threshold level (Bainbridge et al., 2015). Lessons from these studies, along with new data generated in adult animal models, will help better inform the optimal treatment conditions that will maximize successful hearing outcomes.

While equivalent data do not yet exist in human inner ear gene therapy, preclinical animal studies have indicated secondary benefits associated with long-term hearing restoration. A recent study by Nist-Lund et al. (2019) examined the breeding efficiency and survival in a mouse model of recessive *TMC1* deafness. Animals injected with AAVs carrying *Tmc1* transgene at P1 not only showed improvements in auditory function and balance behavior, but also produced higher number of litters with significantly higher survival rates and near-normal growth rates. Follow-up studies should not only continue to examine the treatment effects of restoring gene expression at longer time points, but also probe the dynamics of gene expression and effects on normal inner ear development.

A potential shortcoming of AAV-mediated gene delivery is the limited cargo size of approximately 4.7–5 kb. As such, delivery of large gene sequences using viral vectors can be challenging. This problem can be partially overcome by dual-AAV systems, where each of the two AAV vector carries a fragment of the large transgene and the two vectors are reassembled to reconstitute the full-length expression cassette in the target cell (Ghosh et al., 2008). Two recent studies used split viral vectors carrying otoferlin cDNA in otoferlin knock-out mice. This led to dual transduction in nearly half of the target IHCs, restoration of protein expression to 30% of wild-type levels, and partial rescue of auditory function (Akil et al., 2019; Al-Moyed et al., 2019). These results provide proof-of-concept evidence that large gene constructs can be transduced to the cochlear sensory epithelium to at least partially restore hearing in animals. The use of different AAV serotypes and variations in injection timing and dosage may further optimize outcomes in the future.

### Non-viral Approaches

Non-viral vectors, including liposomes, polymeric nanoparticles, and synthetic peptides, offer a powerful alternative strategy for delivery of therapeutics to the inner ear. Compared to viruses, nanomaterials have several distinct advantages. They can be engineered to precisely target a specific cell subpopulation, exhibit low immunogenicity and toxicity, and multiplex in a high-throughput manner to simultaneously address multiple gene targets and pathways. Nanotechnology tools have been employed extensively in fields such as cancer therapeutics to improve the efficacy and accuracy of delivery of small molecules, biologics, and nucleic acid drugs. Nanomaterial-based cochlear delivery systems using liposomes, peptides, or polymers have been developed in recent years and have shown limited success in applications including genetic hearing loss.

Liposomal agents consisting of cationic lipids, which form a bi-layered structure that protects nucleic acids from degradation and antibody neutralization, have enabled cytosolic delivery of nucleic acids into the cytosol for therapeutic applications. Delivery of cre-recombinase and genome editing agents by lipid complexes resulted in 90% recombination and 20% genomic editing in neonatal mouse OHCs *in vivo* (Zuris et al., 2015). Gao et al. (2018) recently developed cationic lipid nanoparticles that encapsulated CRISPR/Cas-9 complexes targeting the *Tmc1^Bth^* allele. In a mouse model of dominant genetic deafness, injection into the cochlea of neonatal mice resulted in moderate reduction of progressive hearing loss and improved hair cell survival *in vivo* (Gao et al., 2018). Of note, Cas9-single guide RNA was coupled to the cationic lipid formulation, Lipofectamine 2000^®^, an unmodified commercially available lipid transfection reagent that does not have any specificity toward a cell subpopulation. As many commercial lipid-based reagents have been shown to mediate significant non-specific modulation of gene expression and cytotoxicity, further work is needed to optimize the efficacy of delivery and minimize off-target effects (Lv et al., 2006). Furthermore, positively charged lipid nanoparticles could evoke a dramatic pro-inflammatory response marked by upregulation of Th1 cytokines, such as IL-2, IFN-γ, and tumor necrosis factor alpha (TNF-α), when administered systemically into mice (Kedmi et al., 2010). While therapeutics delivered to the cochlea are thought to remain localized to the inner ear and isolated from systemic circulation by the BLB, any immunostimulatory effects through potential systemic exposure could significantly confound the experimental results. Future studies must carefully assess and minimize the effects from off target immune stimulation while maintaining the efficiency and tissue specificity of delivery. Chemical modifications to lipid molecules could be designed in a rational fashion using computational approaches. Furthermore, high-throughput screening technologies of large combinatorial libraries of lipid-like materials should be utilized to identify *in vivo* carriers that specifically target hair cells or supporting cells in the organ of Corti (Whitehead et al., 2014).

A distinct advantage of nanoparticle systems is their “tunability” toward a specific delivery need. By modifying the surface chemistry and altering biophysical properties of nanomaterials, one can optimize inner ear bioavailability, reduce clearance, and improve drug targeting. Polymers such as chitosan-glycerophosphate that slowly degrade over time have been customized to enable the sustained-release of dexamethasone and gentamicin into perilymph over several days in mice (Lajud et al., 2015), and deliver a small molecule to protect mice from noise-induced hearing loss (Kayyali et al., 2018). Elsewhere, targeting ligands such as peptides improved the specificity of nanoparticles toward OHCs (Surovtseva et al., 2012; Kayyali et al., 2018), spiral ganglion cells (Roy et al., 2010), or cochlear nerve cells (Zhang et al., 2011).

Recently, a new class of peptides carrying short interfering RNAs (siRNA) against TNF-α, known as tumor-penetrating nanocomplexes, was used to actively target primary vestibular schwannoma cultures *in vitro* (Ren et al., 2017). These nanoparticles are based on a new class of tissue-targeting and tumor-penetrating peptides, where the RGD-motif bind to integrins overexpressed on the surface of cancer cells and endothelial cells and subsequently undergo proteolytic processing to unveil a cryptic RXXR domain, which ultimately increases tissue permeability and enables translocation of delivered cargo (Ruoslahti et al., 2010; Liu et al., 2017). By engineering a peptide consisting of a tandem tumor-penetrating and membrane-translocating/siRNA-binding domain, the resulting nanoparticles can penetrate tumor tissue when administered systemically. This platform holds great promise to gain access across the BLB, by increasing the permeability through barriers such as the RWM or oval window. Future work should be directed toward utilizing these novel tumor-penetrating nanomaterials to optimize the *in vivo* delivery of gene editing or gene silencing agents through the RWM.

## Surgeries for Gene Therapy Delivery

To obtain surgical access to the inner ear in patients, two principal approaches employed clinically include intra- or trans-tympanic delivery, where the therapeutic is instilled into the middle ear space and allowed to diffuse into the inner ear; and intracochlear delivery, where the drug is introduced directly into the inner ear via the RWM, the oval window, or a semicircular canal (Figure 1A). An effective method of delivery suitable for clinical translation should not require extensive surgical dissection while providing sufficient access to the organ of interest in a minimally invasive fashion. If the patient has any residual hearing, the delivery approach should try to preserve it as much as possible. While intravenous or intraperitoneal injections have been the gold standard for treatment of disease processes such as cancer or bacterial sepsis, the unique BLB surrounding the cochlea may make it difficult for drugs to reach the inner ear. Procedures for local delivery employed in animal models include cochleostomy in the lateral wall of the cochlear basal turn, canalostomy in a semicircular canal, or injection through the RWM; while recent studies have shown general safety of these procedures, they have also highlighted the risk of inner ear injury and permanent SNHL. In this section, we review various routes of application of gene therapy agents with a focus on practical applications in patients.

Intratympanic or transtympanic injections are considered generally low risk procedures to access the middle ear space. In adult patients, this can be done under topical anesthesia in the office, where a small portion of the tympanic membrane is anesthetized and up to 0.7 mL of drug can be instilled through the perforation into the middle ear. While technically simple to perform, the drug must remain in contact with the RWM for a prolonged period of time to allow enough drug to cross the RWM (Salt and Plontke, 2009). Equilibration and distribution of the drug toward other middle ear spaces or through the Eustachian tube can lead to significant losses, which can be partially overcome by either sustained release or multiple injections over time. The patient may experience minor temporary discomfort associated with the injection, and rare complications may include otorrhea or persistent TM perforation.

Another approach to access the inner ear is directly through the oval window. Normally, the stapes footplate overlies the oval window, and a mucosal epithelium similar to RWM lines the footplate facing the vestibule (King et al., 2013). In animal models, a significant but variable portion of intratympanically administered small molecule drugs were found to enter the scala vestibuli via the stapes footplate. Gadolinium contrast was found at higher levels in the vestibule than scala tympani after middle ear application. Using quantitative volumetric simulations, up to 90% of gadolinium entered the vestibule through the oval window rather than the RWM in guinea pigs (King et al., 2011). Interestingly for gentamicin, 35% of the drug entered the perilymph via the stapes and its annular ligament, which resulted in higher drug concentrations in the scala vestibuli than scala tympani due to a slower rate of elimination (Salt et al., 2016). In human cadaveric temporal bones, bisphosphonate introduced via the oval window could reach the apical turn of the cochlea (Kang et al., 2016). In patients with otosclerosis, a bone remodeling disease of the otic capsule and one of the most common causes of acquired hearing loss, access to the vestibule and scala vestibuli is possible during stapedotomy where the stapes footplate is fenestrated. Therefore, by incorporating the drug of interest into the prosthesis, the therapeutic can be released in a controlled fashion into the scala vestibuli. The U.S. gene therapy trial of Ad5.GFAP.Hath1 for the treatment of non-genetic hearing loss delivers the viral vector *via* a stapedotomy.

A direct method for accessing the RWM can be achieved through a transcanal surgical procedure. With the advent of novel surgical technologies, it is now possible to directly access the round window niche (RWN) through a transcanal approach in the vast majority of patients using an endoscope. Endoscopic ear surgery not only enables superior visualization of complex landmarks within the middle ear (Figure 1B) and mastoid, but also allows specialized, angled instruments to access structures through a transcanal approach (Tarabichi and Kapadia, 2016). In a recent series on round window anatomy, temporal bone studies demonstrated that there is a consistent transcanal angle to reach the RWM perpendicularly through the external auditory canal (EAC), which measured approximately 115-degrees (Fujita et al., 2016). Importantly, one of the key hurdles to reaching the true RWM is obstruction in the RWN by a pseudomembrane, as 72% of temporal bones showed partial or full obstruction, as well as bony overhangs of median length of 1.77 mm which limit the direct access to the RWM (Fujita et al., 2016). In this series, nearly a third of specimens required drilling to expose the entire RWM from the EAC. Therefore, in future studies on cochlear drug delivery, it is critical to ensure that the RWN is drilled away or filled with a drug after removal of the pseudomembrane so that the drug is in contact with the entirety of the RWM.

The RWM is a three-layered structure lined by squamous epithelium sandwiching a connective tissue layer, which serves as a dynamic barrier that protects the inner ear. A locally administered drug must permeate through the RWM to reach the perilymph within the scala tympani. The permeability of the RWM can be influenced by particle size, charge, and concentration (Goycoolea, 2001), surgical manipulations such as air suctioning nearby (Mikulec et al., 2008), and endotoxins (Ikeda and Morizono, 1988). Conditions such as otitis media that promote middle ear inflammation could also increase RWM permeability through the regulation of cytokines and tight junction proteins (MacArthur et al., 2013). Strategies to artificially enhance transport across the RWM by prolonging the time of exposure to the drug included the use of a gelatin sponge (Jero et al., 2001), thermoreversible hydrogels (Honeder et al., 2015), partial digestion with collagenase (Wang et al., 2012), co-treatment with hyaluronic acid (Shibata et al., 2012), or microperforations (Kelso et al., 2015).

Despite improvements in surgical instrumentation and manipulation of the RWM, the bioavailability of intratympanically administered therapeutics is still remarkably low. In guinea pigs, only 2.5% of gentamicin was present in the cochlear basal turn when it was irrigated across the RWM for nearly 3 h, and only 0.17% when the bulla was instilled with the drug for 2 h (Mikulec et al., 2008). Unfortunately, it is nearly impractical to translate these protocols into clinical use due to the lengthy nature of the protocol. Furthermore, some gene vectors that are designed to efficiently transduce cells of the auditory sensory epithelium *in vitro* may need to be placed in the scala media to be effective. In CI, a growing area of investigation involves using the implant electrode as a conduit for intracochlear delivery of drugs that may rescue hair cell or neuronal damage for hearing preservation. This could be achieved either through incorporation of the drug into the electrode, or co-administration of the therapeutic at the time of cochleostomy. Implantation of electrode arrays coated with fibroblasts over-expressing brain-derived neurotrophic factor (BDNF) or neurotrophin 3 (NT3) may have a protective effect on SGNs (Rejali et al., 2007; Richardson et al., 2009; Pfingst et al., 2017). Pre-treatment with an anti-apoptotic molecule targeting the MAPK/JNK pathway shortly before electrode insertion could protect sensory epithelium in the organ of Corti from insertion trauma and preserve hearing thresholds in guinea pigs (Eshraghi et al., 2013). CI electrodes capable of eluting dexamethasone have been shown to preserve residual hearing and reduce insertion trauma in animal models (Douchement et al., 2015), which may be through reduction of cochlear fibrosis, suppression of local immune reactivity, and global changes in gene expression (Farhadi et al., 2013; Takumi et al., 2014; Wilk et al., 2016). In addition, a phase II multicenter trial in Europe was recently completed to assess the safety and efficacy of intratympanic steroids during CI (EudraCT Number: 2015-002672-25). Future implants may be optimized through the incorporation of gene delivery vectors targeting sensory cells within the scala tympani or SGNs.

## Technical Considerations

The human middle ear and inner ear are both exquisitely well-balanced systems so that small perturbations of the microenvironment, such as the introduction of drugs and their respective delivery agents through microsurgical manipulations, could have significant effects on the homeostasis of the entire system. While animal models offer invaluable insight in predicting drug pharmacokinetics and pharmacodynamics for preclinical studies, the translation into clinical testing in patients typically presents with additional challenges owing to differences in the volume and anatomy of inner ear organs. The cochlear aqueduct, a bony channel that projects from the posterior fossa to the cochlear basal turn (Figure 1A), is typically obliterated in humans but widely patent in rodents. The patency of the duct has been attributed to one of the mechanisms by which drugs reach the contralateral ear in the so-called “Schreiner effect” (Stover et al., 2000). Sampling of perilymph via the RWM in rodents could lead to contamination with CSF across the cochlear aqueduct at a significant rate of 0.5–2 μL/min (Hirose et al., 2014). Rodent perilymph samples >5 μL could contain as high as 80% of CSF in guinea pigs (Salt et al., 2003). Therefore, perforation of the RWM could result in artifactual CSF flow near the basal turn, thereby displacing the drug within minutes of application. This loss can be greater if the site of injection is not completely sealed. As such, one must consider both the technique of injection and any attempts in preventing contamination when evaluating the efficacy of inner ear drug delivery agents.

In addition, the effective concentration of the drug is likely also dependent on the rate and volume of injection into the perilymph. A low rate of injection (100 nL/min) over a prolonged period of time is required to drive the drug into perilymph while minimizing traumatic perturbations to the inner ear (Salt and Rask-Andersen, 2004). In guinea pigs, without a proper seal around the injector, leakage of perilymph around the pipette resulted in wash-out of the drug by over 60% at a rate of approximately 0.09 μL/min. This loss could be mitigated by the application of hyaluronate gels over the RWM (Salt et al., 2007). Recently, Salt et al. (2017) investigated the concentration gradient along the scala tympani of a model drug (FITC-dextran) delivered via a pump incorporated into a cochlear implant in guinea pigs. A significant concentration gradient was observed despite a prolonged duration of injection of 24 h, and fluid leakage at the site of cochleostomy could lead to significant drug wash-out (Salt et al., 2017). These results highlight the importance of technical considerations when delivering drugs to the inner ear. In patients, the volume of scala tympani is significantly larger than rodents, and small volume perturbations will likely have less impact on the distribution of drugs. Furthermore, the patency of the cochlear aqueduct is reduced, and CSF pressure is typically negative when the patient is sitting when procedure is performed in the clinic. As a result, displacement of the drug either via CSF influx or leakage through cochleostomy may occur at a substantially lower rate in humans than rodents. Nevertheless, a systematic, quantitative approach is paramount when designing delivery systems for clinical applications to achieve reliable and consistent results.

## Future Outlook

Since the first human gene therapy treatment in 1990, there have been 2930 gene therapy clinical trials that have been completed, were ongoing, or clinically approved world-wide. Over two-thirds of the trials were conducted in the United States (Hanna et al., 2016). There is also a steady increase in the number of newly approved/initiated trials over time, with 163 trials in 2015 alone and nearly 600 more trials since 2015. Adeno-associated viral vectors were utilized in 8.1% of the trials to date^2^.

Currently, over 20 clinical trials for hearing loss therapies are ongoing in the United States with six potential therapeutic molecules; one of these trials involves gene therapy. There are over 80 active trials in Europe, Asia, and Australia with many more candidate drugs being actively investigated. Preliminary results should be available within the next 2 years, and other platforms are currently being tested in early clinical studies with numerous drugs on the horizon. If early results meet the specified efficacy endpoints, the inclusion criteria will likely see an expansion to other patient populations such as children with early or congenital SNHL. On the contrary, if results from these trials do not meet criteria in the treated population, meticulous follow-up studies must be carried out to determine the cause of failure, which may include patient stratification, route of delivery, and measurement of outcomes. Ultimately, the successful translation of a novel therapeutic from the laboratory bench to the otology clinic or operating room require a multidisciplinary approach through the collaboration between molecular biologists, virologists, chemists, biomedical engineers, otologic surgeons, government, and business leaders.

As treatments for hearing loss become more personalized, more gene targets that are amenable to targeting will be uncovered. Gene therapy can involve not only the insertion of a transgene through efficient viral transduction, but also silencing of a dominant negative allele through miRNA or siRNAs. Off-target effects will be minimized through enhancing the specificity of therapy. Next-generation CRISPR-Cas systems will be harnessed for precise disruption and editing of DNA or RNA for each patient.

The efficacy and specificity of the gene delivery agent will also likely improve. Engineering of the capsid proteins may further improve viral tropism so that viruses can be tuned to preferentially target the cell subpopulation in the sensory epithelium while minimizing off-target effects. Incorporating cell type-specific promoters will enable the precise targeting and gene expression in cellular subpopulations within the cochlea. Simultaneous testing in animal models and humans will highlight differences between the two, and obstacles such as viral immunogenicity can be addressed. Active, or membrane-penetrating delivery platforms may provide a method to traverse cellular tight junctions (Ruoslahti, 2017). This may significantly enhance the bioavailability of the drug if hearing is not impaired. Finally, deployment of “smart,” on-demand drug delivery systems may further improve drug availability by fine-tuning the release kinetics and minimizing drug loss.

Future surgical innovations and technologies could help better detect electrophysiological changes in the inner ear associated with therapeutic interventions. Electrocochleography studies could provide insight into cochlear changes in real time during an implant and predict hearing thresholds postoperatively, and may be utilized to monitor inner ear physiology and minimize the risk of cochlear damage during electrode insertion or infusion of gene delivery vectors (Dalbert et al., 2018). More sophisticated electrodes are being developed to incorporate neuroprotective substances and drugs. The incorporation of microsurgical and robotic tools in otologic and neurotologic surgeries could make surgeries more precise, less traumatic, and customized to each patient’s unique anatomy.

Current assessments of hearing levels largely rely on indirect measurements such as audiograms, otoacoustic emissions, or auditory brainstem responses. *Post mortem* temporal bone histology has been the gold standard to visualize the pathology underlying hearing loss. However, the advent of intracochlear imaging has been challenging due to the complex three-dimensional anatomy and complete encasement in the bony otic capsule. Future development of tools to non- or minimally invasively assess the function of the inner ear at a cellular resolution will likely revolutionize the way hearing loss is diagnosed and treated. Optical coherence tomography (Dong et al., 2018), micro optical coherence tomography (Iyer et al., 2016), and two photon fluorescence imaging (Yang et al., 2013) have the potential to visualize cochlear microanatomy at high resolution, thus overcoming limitations of bright field endoscopy (Chole, 2015). While synchrotron radiation phase contrast imaging reveals intracochlear microanatomy through the encased bone, future work is needed to minimize radiation energy before this technology can be translated to the clinic (Iyer et al., 2018). In the meantime, light sheet microscopy (Santi, 2011) and incorporation of calcium- or potassium-based molecular imaging markers may shed additional light on the integrity and function of hair cells and other cochlear cells in real time. Together, by taking an interdisciplinary approach and combining new genomic data, better bioengineering tools, and innovative surgical approaches, clinical success for gene therapy for SNHL and inner ear disease is not far away.

## Author Contributions

YR, LDL, and KMS designed the study and conducted the research and literature search. YR and KMS prepared the manuscript. All authors edited and approved the final manuscript.

## Conflict of Interest Statement

The authors declare that the research was conducted in the absence of any commercial or financial relationships that could be construed as a potential conflict of interest.

## Footnotes

1 http://hereditaryhearingloss.org/

2 http://www.abedia.com/wiley/vectors.php
